# Single- and Twin-Photons Emitted from Fiber-Coupled Quantum Dots in a Distributed Bragg Reflector Cavity

**DOI:** 10.3390/nano12071219

**Published:** 2022-04-05

**Authors:** Xiangjun Shang, Shulun Li, Hanqing Liu, Xiangbin Su, Huiming Hao, Deyan Dai, Xiaoming Li, Yuanyuan Li, Yuanfei Gao, Xiuming Dou, Haiqiao Ni, Zhichuan Niu

**Affiliations:** 1 State Key Laboratory for Superlattices and Microstructures, Institute of Semiconductors, Chinese Academy of Sciences, Beijing 100083, China; xjshang@semi.ac.cn (X.S.); lishulun@semi.ac.cn (S.L.); hqliu@semi.ac.cn (H.L.); suxb@semi.ac.cn (X.S.); hmhao@semi.ac.cn (H.H.); dydai@semi.ac.cn (D.D.); xmli@semi.ac.cn (X.L.); xmdou04@semi.ac.cn (X.D.); nihq@semi.ac.cn (H.N.); 2 Beijing Academy of Quantum Information Sciences, Beijing 100193, China; yyli@semi.ac.cn (Y.L.); yfgao@semi.ac.cn (Y.G.); 3 Center of Materials Science and Optoelectronics Engineering, University of Chinese Academy of Sciences, Beijing 100049, China

**Keywords:** InAs quantum dot, fine structure splitting, thermal stress, light hole level, photon pair, polarization correlation, single-mode fiber coupling

## Abstract

In this work, we develop single-mode fiber devices of an InAs/GaAs quantum dot (QD) by bonding a fiber array with large smooth facet, small core, and small numerical aperture to QDs in a distributed Bragg reflector planar cavity with vertical light extraction that prove mode overlap and efficient output for plug-and-play stable use and extensive study. Modulated Si doping as electron reservoir builds electric field and level tunnel coupling to reduce fine-structure splitting (FSS) and populate dominant XX and higher excitons XX^+^ and XXX. Epoxy package thermal stress induces light hole (lh) with various behaviors related to the donor field: lh h_1_ confined with more anisotropy shows an additional X_Z_ line (its space to the traditional X lines reflects the field intensity) and larger FSS; lh h_2_ delocalized to wetting layer shows a fast h_2_–h_1_ decay; lh h_2_ confined shows D_3h_ symmetric higher excitons with slow h_2_–h_1_ decay and more confined h_1_ to raise h_1_–h_1_ Coulomb interaction.

## 1. Introduction

**Figure 1:**
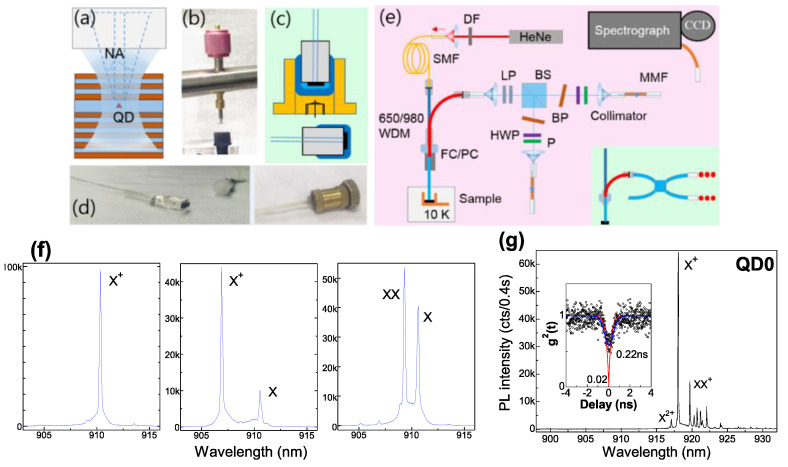
(**a**) Schematic fiber coupling of a QD in a planar DBR cavity; (**b**) spring pressure at QD chip backside for bond; (**c**) model and (**d**) real image of epoxy package (dark blue) and copper mount (yellow), cured ultraviolet adhesive (light blue) as a stress buffer, and a ceramic ferrule as fiber interface; (**e**) setup for PL spectroscopy and photon correlations, inset: fused SM fiber BS (780HP, Thorlabs) sometimes used; (**f**) PL spectra of single-QDs on sample: (**left**): intrinsic, (**middle**): near hole defects, (**right**): near Si donors; (**g**) PL spectrum of fiber-coupled single QD, QD0 with a dominant X^+^ and higher XX^+^ and X^2+^; inset: X^+^ auto-correlation with theoretical (red) and convoluted (blue) fitting.

Semiconductor quantum dots (QDs) have been identified as a promising solid-state quantum emitter with feasible integration of micro-cavities and enhanced coupling to light as compared to real atoms. At stronger pump, several excited carriers presented in a QD lead to multi-exciton states with sharp spectral lines separated energetically by the confinement potentials and Coulomb configuration interactions. Each can be used as a single-photon emitter, and a definite photon-pair emission is built in them. However, QD light extraction relies on microscope optics with fine tuning; its coupling in single-mode (SM) fiber with core diameter (D_M_) of 2–9 μm [1] for practical use—e.g., time-modulated two-photon interference [2] or inter-QD interference [3]—is usually made by aspheric lens. A direct near-field fiber bond realizes integrated single-QD emitters for a plug-and-play stable use for extensive study. Instead of tapered fiber evanescent lateral coupling [4,5] or cleaved fiber facet vertical coupling [6,7,8] with QD host precisely positioned to the fiber core, an efficient vertical coupling of a QD at wavelength (λ) ~0.9 μm is proved by random bond of V-groove fiber array with large smooth facet and no bend (i.e., angle self-aligned) to QD chip with large-area low-density InAs/GaAs QDs in a distributed Bragg reflector (DBR) cavity for vertical light extraction [9], with coupling efficiency mainly dependent on the cavity; a >3-fold enhancement of fiber-output single-photons has been achieved in an optimal pillar cavity with an intrinsic radiative lifetime < 0.2 ns (Purcell factor > 3) [10]. This work presents our resent study on fiber devices: (1) a planar DBR cavity with only fundamental cavity mode (CM) is bonded to Nurfern 780HP SM fiber (numerical aperture (NA) ~0.13, D_M_ ~4.4 μm) for optical mode overlap (Figure 1a), with single QD selected by small D_M_ and NA, enabling flexible SM-fiber selection (especially D_M_ ~2 μm); (2) modulated Si doping as electron reservoir [11] builds electric field and level tunnel coupling [12] to reduce fine-structure splitting (FSS) and populate dominant X and XX (in pair rate ~12 Mcps) and higher excitons XX^+^ (2e_1_2h_1_1h_2_), XXX (2e_1_2h_1_1e_2_1h_2_), XXX^+^ (2e_1_2h_1_1e_2_2h_2_) and XXXX (2e_1_2h_1_2e_2_2h_2_); (3) epoxy thermal stress induces light hole (lh) h_1_ and h_2_ with various distribution in the donor field to affect exciton symmetry, FSS, Coulomb interaction and inter-level decay. The fiber-output XX-X pairs are promising for polarization correlation.

## 2. Materials and Methods

Dilute InAs QDs are grown in epitaxy on semi-insulating GaAs (001) substrates with a gradient indium flux and subcritical deposition amount [13] and integrated in a planar GaAs/Al_0.9_Ga_0.1_As DBR cavity with CM at 910~920 nm (Q~1300). As Figure 1f indicates, QDs with no donor show a dominant X^+^ from background p-impurity; hole traps induce a secondary X by a slow tunnel capture [14]; delicate modulated Si doping added above QDs populate dominant X and XX. Single-QD region on chip is pre-selected by temperature (T) ~5 K micro-photoluminescence (PL) spectroscopy [15]; the fibers with single-QDs are post-selected by T < 20 K PL spectroscopy by a fused SM fiber 650/980-nm wavelength division multiplexer (WDM, SM28e). For no-space bond, a pressure is added (Figure 1b) during the ultraviolet adhesive (Norland61) curing; bond separation is avoided by epoxy package in copper mount (Figure 1c,d) that remains thermal stress (see Table 1, thermal expansion coefficient, very large for cured epoxy); cured ultraviolet adhesive acts a stress buffer to QD chip. PL spectroscopy is by spectrograph with a low-noise CCD under HeNe laser continuous-wave (cw) pump, power tuned by density filter (DF). Figure 1e illustrates the Hanbury-Brown and Twiss (HBT) setup to measure correlations: fiber device PL output connects a non-polarized 50:50 beamsplitter (BS) after λ > 900 nm longpass (LP) set (optical density, OD > 12 at the laser 633 nm and >4 at the matrix PL, 800~870 nm); exciton lines are filtered by narrowline bandpass (BP) (window λ < 930 nm, tunable by tilt angle, Δλ < 0.5 nm); multi-mode fiber (MMF) with collimator for collection and Si-avalanched photodiode (APD) detectors for photon count; auto/cross-correlations g^2^(t) between the two detectors are fitted with convolution of the HBT system response function (Gaussian, approximately [16]) for decay time analysis; polarization is selected by a half-wave plane (HWP) or quarter-wave plane (QWP) and a linear polarizer (P), also used to observe FSS oscillation deduced from time-integrated PL spectra [11] and unaffected by bi-refraction in fiber. For pulsed pump, a 640 nm diode laser with repeat rate ~80 MHz and pulse width ~70 ps is used. The radiative lifetime and extraction efficiency are usually measured/estimated under pulsed pump for QDs in flat band. For QD coupled to electron reservoir, the tunnel population is different under cw and pulsed pumps: as seen below, under pulsed pump, higher excitons are populated with XX and X emission efficiency reduced; under cw pump, dominant X and XX populated can be considered as a pure three-level ladder system to study the radiative lifetime and extraction efficiency. Under above-band pump, QD emission includes population and radiation. The exciton intrinsic radiative lifetime is estimated by the decay time in photon correlation under saturation with dense carriers in the barrier for a rapid population. XX with 2e_1_2h_1_ and independent transitions of two e-h pairs shows an intrinsic radiative lifetime near half that of X, while X^+^ has the same one as X. As our previous measure of auto-correlations of a dominant X^+^ shows [12], X^+^ decay time under saturation is 0.25 ns, reflecting X^+^ (X) intrinsic radiative lifetime in a DBR cavity with >3-fold Purcell enhancement. Here, QD-fiber device, QD0 undoped, in Figure 1g also shows a dominant X^+^ with saturated decay time ~0.22 ns and g^2^(0) ~0.02 (i.e., pure single photon). For QDs coupled to donor levels, the tunnel population is usually dependent on the pump power [12], i.e., electron density in the coupled level. A half-filled coupled level under cw pump shows XX (2e) population time nearly twice that of X (1e) while under pulsed pump with electron reservoir temporally saturated and the coupled level full-filled; X (1e) and XX (2e) tunnel population show nearly the same time. Under saturation with XX and X in comparable intensity, X and XX decay times in auto-correlations are used to estimate their intrinsic radiative lifetimes; the decay time of bunching peak in their cross-correlation reflects the difference of their radiative lifetimes. The fiber extraction efficiency is estimated with dominant XX and X spectral lines under cw pump. This work presents three single-QD fiber devices, QD1~3 with donor fields and level coupling, for illustration. A well selection of single QD (i.e., multi-excitons from the same QD) is reflected. The donor field intensity is well characterized by lh h_1_ and h_2_ formed in epoxy package thermal stress; their various distribution in the donor field show lh excitons with different FSS, symmetry, h–h Coulomb interaction and inter-level decay with physical understanding.

## 3. Results

### 3.1. Electron Level Coupling and Stress-Induced lh Levels

**Figure 2:**
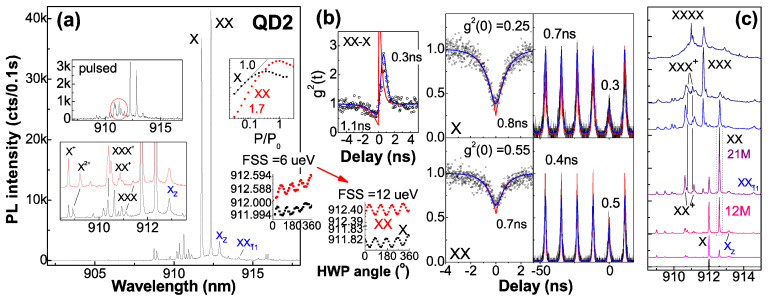
(**a**) PL spectra of single-QD fiber, QD2 in donor field with level coupling. Insets: spectrum under pulsed pump; spectra in 2nd (red) and *N*-th (black) cryogen circles with shift of X^2+^, XX^+^, and XXX; sharper X_Z_ and raising FSS from 6 to 12 μeV; intensity pump power dependence. (**b**) Photon auto-/cross-correlations under cw and pulsed pumps with theoretical (red) and convoluted (blue) fitting, decay times given; FSS oscillation of XX and X. (**c**) Cw pump power-dependent spectra.

**Figure 3:**
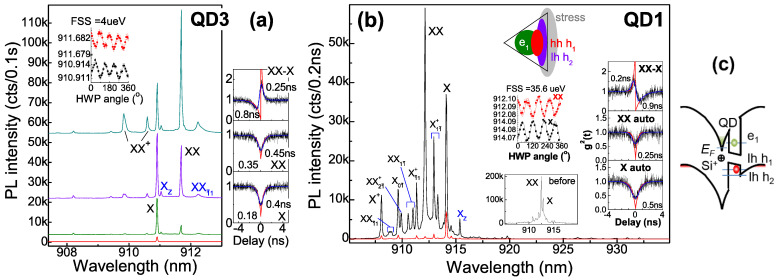
PL spectra of single-QD fibers, (**a**) QD3 in lower donor field with small FSS and (**b**) QD1 in stronger field with large FSS and negative E_B_ enlarged by stress. (**c**) Schematic e and h distribution. QD1 in stress field with e and h confined for more overlap shows X and XX with shorter decay time and higher excitons in D_3h_ symmetry (confined lh h_1_ and h_2_, model in inset). Insets: FSS oscillation, photon correlations with similar fitting, QD1 spectrum before epoxy coverage.

Figure 2 presents QD2. The auto-correlations show nearly the same decay times for X (0.8 ns) and XX (0.7 ns) under cw pump, while 0.4 ns corresponds to XX under pulsed pump with photoelectrons temporally saturated in reservoir (which is half that of X—0.7 ns); as the decay time of the bunching peak reflects, the difference of X and XX radiative lifetimes is 0.3 ns, so X and XX show intrinsic radiative lifetimes of 0.6 ns and 0.3 ns, respectively, with the remaining 0.2 ns and 0.4 ns representing 1e and 2e tunnel population time under cw pump with the coupled level half-filled, while 0.1 ns corresponds to 1e and 2e tunnel under pulsed pump with the coupled level full-filled. X intrinsic radiative lifetime ~0.6 ns, longer than X^+^ ~0.25 ns in a DBR cavity, reflects delocalized wave function in the coupled level for slow e-h transition. By fitting experimental data under pulsed pump with double exponential functions, the higher auto-correlation g^2^(0) (multi-photon probability)—0.3 for X and 0.55 for XX—are obtained, nearly the same under cw pump, due to recapture from reservoir [17], in contrast to near zero g^2^(0) for a dominant X^+^ in flat band (QD0 in Figure 1g). In the non-pulse region of auto-correlation, the higher background count in X also reflects a fluent 1e recapture. In fact, QDs here (include QD3 and QD1 in Figure 3) show a prior X appearance under weak pump in power dependence slope of 1.0 and XX in slope of 1.7 (i.e., 1e filling in the coupled level), unlike a prior X^−^ in [11] (i.e., 2e filling due to Fermi level pin by a closer donor); the donor field is a little higher to get the minimal FSS: QD3 in a lower field has FSS ~4 μeV while QD2 and QD1 in higher fields show higher FSS. There is an additional X line, X_Z_, from lh h_1_ polarized in *z*-axis coexisting with the traditional X lines (power-dependent spectra in Figure 2c and that of QD3 and QD1 in Figure 3), similar to a strain-tuned GaAs QD [18]; the energy offset between X_Z_ and the traditional X lines reflects e-h separation, Stark shift and donor field intensity: 0.18 meV in QD3 (Figure 3a) in a lower field and 1.68 meV in QD2 (Figure 2) and 1.89 meV in QD1 (Figure 3b) in a higher field, consistent with monolithic increase of FSS from ~4 μeV in QD3 to ~6–12 μeV in QD2 and 35 μeV in QD1, due to lh h_1_ more confined with more anisotropy in the donor field. In contrast, lh h_2_ is more delocalized: in QD3 in a lower field with lh h_2_ coupled to wetting layer, a fast h_2_–h_1_ decay is expected and XXī_1_ (2e_1_1h_1_1h_2_) shows considerable intensity in a broad linewidth from a fast h_2_–h_1_ decay of its transition target X_01_ (1e_1_1h_2_); QD1 in a much stronger field with lh h_2_ decoupled from wetting layer and confined in QD shows higher excitons related to h_2_ such as X_1_ī^+^, Xī_1_^+^ (1e_1_1h_2_1h_2_), X_0_ī, XXī_1_, and XX_1_ī [19]—located around XX [11] with D_3h_ symmetric spectral features and slow h_2_–h_1_ decay, unlike C_2v_ featured X and XX with large FSS ~35 μeV; the slow h_2_–h_1_ decay is likely from their spatial distribution as the model in Figure 3b inset indicates: the stress at QD base with large strain distribution [20] forms lh h_2_ strongly confined there in the donor field and leads to lh h_1_ being more confined (from h_2_ repulsion) for larger V_hh_ and slower h_2_–h_1_ decay; the more confined h_1_ contains more anisotropy for larger FSS in XX and X; in QD2 in a high field with lh h_2_ gradually decoupled from wetting layer by epoxy thermal stress during cryogen circles (see spectra under 2nd and *N*-th cryogen circles, Figure 2a inset), lh h_1_ gets more confined to show increased e_1_–h_1_ overlap for shaper X_Z_, FSS raising from 6 to 12 μeV (i.e., e_1_–h_1_ overlap increased), X^2+^, XX^+^ and XXX blue-shift slightly (i.e., an increased h_1_–h_2_ Coulomb interaction). In QD1 in a stronger field with lh h_1_ more confined, a shape X_Z_, a large FSS and an increased h_1_-h_1_ Coulomb interaction V_hh_ to enlarge negative XX binding energy E_B_(XX) = 2V_eh_ − V_ee_ − V_hh_ [21] as compared to the spectrum before epoxy package (Figure 3b inset, with a slightly negative E_B_(XX) from the field-reduced V_eh_, e_1_–h_1_ Coulomb interaction) are shown. In QD2, under pulsed pump, XX^+^, XXX, and XXX^+^ get relatively stronger (Figure 2a inset), due to rapid tunnel capture of 2e or 3e from reservoir being temporally saturated. In comparison, QD3 shows the highest exciton of XX^+^, small FSS ~4 μeV, and lower auto-correlation g^2^(0)—0.18 for X and 0.35 for XX—due to lower donor reservoir, field, and recapture with nearly the same decay time (XX ~0.45 ns and X ~0.4 ns) under cw pump, shorter than QD2, reflecting excitons with less e–h separation in the lower field and shorter radiative lifetimes—X ~0.25 ns and XX ~0.13 ns—with Purcell enhancement kept; the remaining 0.15 ns and 0.32 ns represent 1e and 2e tunnel times. In QD1 in a stronger electric field, the near zero auto-correlation g^2^(0) for X and XX and their decay times under saturation (X ~0.5 ns and XX ~0.25 ns) reflecting their intrinsic radiative lifetimes, longer than the usual (X ~0.25 ns in a DBR cavity, e.g., QD3 in a lower field) from e–h separation in the field, but smaller than QD2 (X ~0.6 ns), with no tunnel population or recapture due to a large stress field to confine e_1_ and h_1_ and improve their overlap (model in Figure 3b inset). In all, the donor field and level tunnel coupling reduce FSS to ~4 μeV, compared to the usual 20~30 μeV in C_2v_ QDs; unlike lh-heavy hole mixing of h_1_ in a donor field [11], a pure lh h_1_ is formed in the stress field. The donor and stress field modulate lh h_1_ and h_2_ distribution to tune exciton symmetry, FSS, V_hh_, and inter-level decay.

### 3.2. High-Rate Photon Pairs and Polarization Correlation

**Figure 4:**
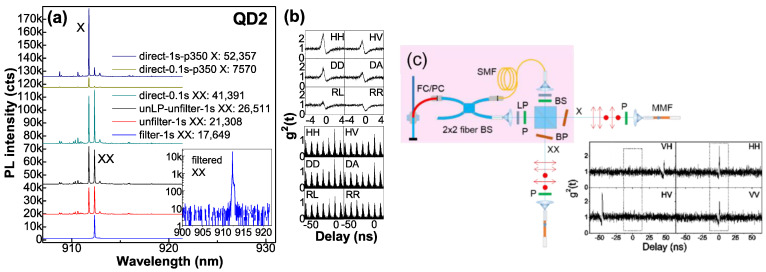
(**a**) PL spectra of QD2 to stimate optical route efficiency. The intensity ratio for integrated time 0.1 s and 1 s is estimated by X peak intensity under low pump p350, i.e., 52,357/7570 = 6.9 (less than 10 due to CCD processing time), which has been checked for many QDs, so the direct unfiltered spectrum for integrated time of 0.1 s with peak intensity of 41,391 will be 41,391 × 6.9 = 286,276 at peak intensity for integrated time of 1 s. The optical route efficiency is estimated by the filtered XX peak intensity of 17,649 (one beam) and the direct unfiltered one of 286,276, 17,649 × 2/286,276 = 12%. BP efficiency is estimated by BP filtered and unfiltered XX peak intensity: 17,649/21,308 = 83%. LP efficiency is estimated by its filtered and unfiltered XX peak intensity: 21,308/26,511 = 80%. The filtered XX line (inset: semi-log plot) shows photon count rate ~240k cps at APDs, so the overall fiber-output single-photon rate is 240k/17,649 × 286,276/0.33 = 12 Mcps for XX and X in comparable intensity, taking into account Si-APD efficiency. (**b**) Polarization-resolved XX-X cross-correlations under cw and pulsed pumps. (**c**) Post-selection to prepare HH/VV correlation. (**pink region**) Setup: a 2 × 2 fused fiber BS to split light, a SM fiber for delay in one beam, two P orthogonal polarized to select HH (VV) polarized photon pairs in each beam, a BS to group them and separate XX and X for output, filtered by BPs. (**bottom right**) Measurement results of polarization-resolved XX-X correlations with polarization in each output selected by a P. HH and VV show bunching at zero delay while HV and VH show bunching at large delays defined by the delay fiber length, which can be as long as ~km and neglected in time bin selection (dashed rectangular).

The fiber devices prove efficient output. The high-rate XX-X pairs are obtained under cw pump with moderate tunnel population when QD can be considered as a pure 3-level ladder system for efficient quantum-pair emission. To estimate the overall fiber-output photon-pair rate under cw pump, the optical route efficiency is estimated by PL spectral peak intensity of an exciton line in QD2. As shown in Figure 4a, it is 12%, including efficiencies of BP (83%), LP (80%), and MMF collection (18%). When XX and X are saturated with comparable intensity (i.e., ~40,000 cts per 0.1 s of its PL peak intensity), XX is filtered and its single-photon rate is measured at Si-APDs, 240 kcps, corresponding to an overall XX-X pair rate ~12 Mcps, taking into account Si-APD efficiency (33% at λ~900 nm) and the optical route efficiency. As shown in Figure 2c, XX becomes dominant under higher pump, with single-photon rate ~21 Mcps as estimated by PL intensity, the same level as a pillar cavity before 1st lens (20~40 Mcps [22]), reflecting QD at the fiber core center with coupling efficiency > 50%, consistent with simulation [23]. For QD with a dominant X^+^ and radiative lifetime ~0.2 ns (QD0 in Figure 1g), the optimal fiber-output single-photon rate will be the same ~20 Mcps. Figure 4b presents polarization-resolved XX-X correlations in QD2 (FSS ~12 μeV): most clear in HH basis; high in HV, DD and DA from cross-dephasing, carrier scattering or fiber bi-refraction that projects single-photon polarization in H or V to reduce polarization correlation, and nearly zero in RR and RL, reflecting independent HH and VV emissions with little superposition for R and L. The correlations are lower under pulsed pump with the barrier carriers temporally saturated for scattering. For QD3 (FSS ~4 μeV), similarly, the most clear polarization correlation is shown in HH basis; the theoretically predicted entanglement degree is >0.5 [24], but ~0.38 under low pump with fewer barrier carriers for scattering, reflecting fiber bi-refraction to project single-photon polarizations and degrade their polarization correlation, understood through math (α is the phase between H and V):

DD = (H + e^iα^V)(H + e^iα^V)/2 = (HH + e^i2α^VV)/2 + e^iα^HV (1)

DA = (H + e^iα^V)(H − e^iα^V)/2 = (HH − e^i2α^VV)/2

The noisy correlation could be improved by reducing ‘cross-talk’ in HBT setup and back reflection in fibers. To use the fiber-output photon pairs for detection, a post-selection of polarization and time bin is used to recover the HH/VV correlation (see Figure 4c). More desired, H and V polarizations can be kept in polarization-maintaining fiber, PM780HP, for entanglement usage or resonant excitation. The fiber coupling efficiency can be further improved by using SM fibers with tapered facet [25] or high NA [26].

## 4. Conclusions

In this work, we optimize the fabrication of single quantum dot (QD) fiber devices and achieve XX-X pair rate ~12 Mcps for plug-and-play stable use and study. QDs coupled to the donor electric field show smaller FSS and higher exciton population. Epoxy stress-induced light hole (lh) h_1_ confined in QD raises FSS; lh h_2_ delocalized fastens h_2_–h_1_ decay; lh h_2_ confined shows D_3h_ symmetric excitons and more confined h_1_ with slow h_2_–h_1_ decay and large h_1_–h_1_ Coulomb interaction. The combined donor field and stress field to tune lh h_1_ and h_2_ distribution to affect QD exciton behaviors, e.g., FSS, is promising for a physical understanding and a proper design of the hybrid QD structure.

## Figures and Tables

**Table 1:** Thermal expansion coefficient (α) of materials used in fiber bond.

Materials	GaAs	InAs	Al_0.9_Ga_0.1_As	Silica	Cured Epoxy	Copper
α (10^−6^/K)	5.7	4.5	5.2	0.5	57	18

## Data Availability

The data that support the findings of this study are available from the corresponding author upon reasonable request.

## References

[1] Nurfern Fibers. https://coherentinc.force.com/Coherent/specialty-optical-fibers/single-mode.

[2] Ates S., Agha I., Gulinatti A., Rech I., Badolato A. (2013). Improving the performance of bright quantum dot single photon sources using temporal filtering via amplitude modulation. Sci. Rep..

[3] Flagg E.B., Muller A., Polyakov S.V., Ling A., Migdall A., Solomon G.S. (2010). Interference of Single Photons from Two Separate Semiconductor Quantum Dots. Phys. Rev. Lett..

[4] Ahn B.-H., Lee C.-M., Lim H.-J., Schlereth T.W., Kamp M., Höfling S., Lee Y.-H. (2015). Direct fiber-coupled single photon source based on a photonic crystal waveguide. Appl. Phys. Lett..

[5] Daveau R.S., Balram K.C., Pregnolato T., Liu J., Lee E.H., Song J.D., Verma V., Mirin R., Nam S.W., Midolo L. (2017). Efficient fiber-coupled single-photon source based on quantum dots in a photonic-crystal waveguide. Optica.

[6] Muller A., Flagg E.B., Metcalfe M., Lawall J., Solomon G.S. (2009). Coupling an epitaxial quantum dot to a fiber-based external-mirror microcavity. Appl. Phys. Lett..

[7] Cadeddu D., Teissier J., Braakman F.R., Gregersen N., Stepanov P., Gérard J.-M., Claudon J., Warburton R.J., Poggio M., Munsch M. (2016). A fiber-coupled quantum-dot on a photonic tip. Appl. Phys. Lett..

[8] Zolnacz K., Musial A., Srocja N., Große J., Schlosinger M.J., Schneider P.-I., Kravets O., Mikulicz M., Olszewski J., Poturaj K. (2019). Method for direct coupling of a semiconductor quantum dot to an optical fiber for single-photon source applications. Opt. Express.

[9] Ma B., Chen Z.-S., Wei S.-H., Shang X.-J., Ni H.-Q., Niu Z.-C. (2017). Single photon extraction from self-assembled quantum dots via stable fiber array coupling. Appl. Phys. Lett..

[10] Chen Y., Li S.-L., Shang X.-J., Su X.-B., Hao H.-M., Shen J.-X., Zhang Y., Ni H.-Q., Ding Y., Niu Z.-C. (2021). Fiber coupled high count-rate single-photon generated from InAs quantum dots. J. Semicond..

[11] Shang X.-J., Li S.-L., Liu H.-Q., Ma B., Su X.-B., Chen Y., Shen J.-X., Hao H.-M., Liu B., Dou X.-M. (2021). Symmetric Excitons in an (001)-Based InAs/GaAs Quantum Dot Near Si Dopant for Photon-Pair Entanglement. Crystal.

[12] Shang X.-J., Ma B., Ni H.-Q., Chen Z.-S., Li S.-L., Chen Y., He X.-W., Su X.-L., Shi Y.-J., Niu Z.-C. (2020). C_2v_ and D_3h_ symmetric InAs quantum dots on GaAs (001) substrate: Exciton emission and a defect field influence. AIP Adv..

[13] Li M.-F., Yu Y., He J.-F., Wang L.-J., Zhu Y., Shang X.-J., Ni H.-Q., Niu Z.-C. (2013). In situ accurate control of 2D-3D transition parameters for growth of low-density InAs/GaAs self-assembled quantum dots. Nanoscale Res. Lett..

[14] Nguyen H.S., Sallen G., Abbarchi M., Ferreira R., Voisin C., Roussignol P., Cassabois G., Diederichs C. (2013). Photoneutralization and slow capture of carriers in quantum dots probed by resonant excitation spectroscopy. Phys. Rev. B.

[15] Li S.-L., Shang X.-J., Chen Y., Su X.-B., Hao H.-M., Liu H.-Q., Zhang Y., Ni H.-Q., Niu Z.-C. (2021). Wet-etched microlens array for 200 nm spatial isolation of epitaxial single QDs and 80 nm broadband enhancement of their quantum light extraction. Nanomaterials.

[16] Ulrich S.M., Gies C., Ates S., Wiersig J., Reitzenstein S., Hofmann C., Loffler A., Forchel A., Jahnke F., Michler P. (2007). Photon statistics of semiconductor microcavity lasers. Phys. Rev. Lett..

[17] Yu S., Wang Y.-T., Tang J.-S., Yu Y., Zha G.-W., Ni H.-Q., Niu Z.-C., Han Y.-J., Li C.-F., Guo G.-C. (2016). Tunable-correlation phenomenon of single photons emitted from a self-assembled quantum dot. Phys. E Low-Dimens. Syst. Nanostruct..

[18] Zhang J.-X., Huo Y.-H., Rastelli A., Zopf M., Höfer B., Chen Y., Ding F., Schmidt O.G. (2015). Single photons On-demand from light-hole excitons in strain-engineered quantum dots. Nano. Lett..

[19] Karlsson K.F., Oberli D.A., Dupertuis M., Troncale V., Byszewski M., Pelucchi E., Rudra A., Holtz P.O., Kapon E. (2015). Spectral signatures of high-symmetry quantum dots and effects of symmetry breaking. New J. Phys..

[20] Stoleru V.-G., Pal D., Towe E. (2002). Self-assembled (In, Ga)As/GaAs quantum-dot nanostructures: Strain distribution and electronic structure. Phys. E.

[21] Bennett A.J., Pooley M.A., Stevenson R.M., Ward M.B., Patel R.B., de la Giroday A.B., Sköld N., Farrer I., Nicoll C.A., Ritchie D.A. (2010). Electric-field-induced coherent coupling of the exciton states in a single quantum dot. Nat. Phys..

[22] Li S.-L., Chen Y., Shang X.-J., Yu Y., Yang J.-W., Huang J.-H., Su X.-B., Shen J.-X., Sun B.-Q., Ni H.-Q. (2020). Boost of single-photon emission by perfect coupling of InAs/GaAs quantum dot and micropillar cavity mode. Nanoscale Res. Lett..

[23] Shang X.-J., Li S.-L., Ma B., Chen Y., He X.-W., Ni H.-Q., Niu Z.-C. (2021). Optical fiber coupling of quantum dot single photon sources. Acta Phys. Sin..

[24] Hudson A.J., Stevenson R.M., Bennett A.J., Young R.J., Nicoll C.A., Atkinson P., Cooper K., Ritchie D.A., Shields A.J. (2007). Coherence of an Entangled Exciton-Photon State. Phys. Rev. Lett..

[25] OZOptics Shop. https://shop.ozoptics.com/single-mode-taperedlensed-fibers.

[26] Thorlabs Ultra-High NA Fibers. https://www.thorlabs.com/newgrouppage9.cfm?objectgroup_id=340.

